# Loneliness and Problematic Internet Use in Adolescents: The Mediating Role of Dissociation

**DOI:** 10.3390/children11111294

**Published:** 2024-10-25

**Authors:** Alessio Matiz, Fabio D’Antoni, Stefania Pascut, Rebecca Ciacchini, Ciro Conversano, Angelo Gemignani, Cristiano Crescentini

**Affiliations:** 1 Department of Languages and Literatures, Communication, Education and Society, University of Udine, 33100 Udine, Italy; fabio.dantoni@asufc.sanita.fvg.it (F.D.); cristiano.crescentini@uniud.it (C.C.); 2 Department of Psychology, Sapienza University of Rome, 00185 Rome, Italy; 3 Azienda Sanitaria Universitaria Friuli Centrale (ASUFC), 33100 Udine, Italy; 4 WHO Healthy Cities Project, Municipality of Udine, 33100 Udine, Italy; stefania.pascut@comune.udine.it; 5 Department of Surgical, Medical and Molecular Pathology, Critical and Care Medicine, University of Pisa, 56126 Pisa, Italy; rebecca.ciacchini@med.unipi.it (R.C.); ciro.conversano@unipi.it (C.C.); angelo.gemignani@unipi.it (A.G.); 6 School of Advanced Studies, University of Camerino, 62032 Camerino, Italy; 7 Institute of Mechanical Intelligence, School of Advanced Studies Sant’Anna, 56127 Pisa, Italy

**Keywords:** problematic internet use, loneliness, dissociation, mental health, adolescents, mediation analysis, COVID-19 pandemic, high school, students

## Abstract

Background/Objectives. Problematic Internet uUse (PIU) is a multifaceted syndrome characterized by excessive or poorly controlled preoccupations, urges, or behaviors regarding Internet use leading to significant impairments in daily life and mental health. Previous research has separately related PIU to loneliness and dissociation, both in adults and adolescents. The aim of the present study is to analyze the mutual relationship between PIU, loneliness, and dissociation in an adolescent sample, in particular evaluating the indirect effect of dissociation on the relationship between loneliness and PIU. Methods. A cross-sectional design was used with 243 Italian high school students (69.1% females), from year 9 to 13 (age: M = 17.1, SD = 1.4 years), who participated in the study from January to June 2020. They completed measures of PIU (Generalized Problematic Internet Use Scale-2), loneliness (UCLA Loneliness Scale), and dissociation (Adolescent Dissociative Experiences Scale). Socio-demographic and contextual variables were also collected (i.e., age, gender, type of school, school year, sport practice, hobby engagement, assessment before/during the COVID-19 pandemic). Data were analyzed using regression, Pearson’s correlation, and mediation analysis. Results. Severe PIU was observed in 8.6% of the sample. None of the socio-demographic and contextual variables had a significant effect on PIU. Positive medium-sized correlations were observed between PIU, loneliness, and dissociation. Mediation analyses showed an indirect effect of loneliness on PIU through dissociation. Conclusions. Feelings of loneliness may significantly exacerbate adolescents’ PIU by increasing their dissociative tendency. Understanding this dynamic is crucial for developing targeted interventions to address both loneliness and dissociation in efforts to mitigate PIU among adolescents.

## 1. Introduction

Problematic Internet Use (PIU) is the widely used label for a multifaceted syndrome with cognitive, emotional, and behavioral symptoms that significantly interfere with an individual’s ability to manage various aspects of life, including social relationships, academic or work performance, and mental health [1,2,3]. People with PIU may prefer online rather than in-person social interactions, frequently use the Internet as a way to regulate their mood, experience obsessive thoughts about Internet use, and struggle to control or modify their online behavior [4]. PIU can be currently viewed as an umbrella term for a maladaptive pattern of Internet usage [5,6,7,8,9,10,11], which can also include a form of Internet addiction [12,13].

PIU is generally associated with poor mental health [14,15]. Mental health problems such as depression, anxiety, or loneliness can be considered predisposing factors for the development of PIU. Various studies have indeed found that frequent use of the Internet could potentially serve as a way to alleviate boredom, lessen feelings of loneliness and sadness, improve mood, and/or avoid in-person interactions [16,17,18,19,20]. According to the well-recognized cognitive behavioral theoretical model of PIU by Davis [21], which emphasizes that PIU is a generalized phenomenon (i.e., driven by the overall communicative context of the Internet rather than specific online activities), such psychological issues contribute to PIU by making individuals more likely to use the Internet as a coping mechanism.

Among others, the psychological constructs of loneliness and dissociation have been extensively studied in relation to PIU. Both seem to be positively associated with PIU. Loneliness is a feeling of sadness or emptiness resulting from lack of social connection or companionship. When this condition is unattended, it usually has a negative impact on both our mental and physical well-being [22]. A complex and dynamic relationship between loneliness and PIU has been highlighted by reviews [23,24]. For example, it is possible that when PIU is developed it may exacerbate pre-existing loneliness, resulting in a vicious cycle of maintenance of symptoms [25]. Dissociation has frequently been considered a mediator variable in the association between psychological/clinical variables (such as attachment disorganization [26], self-control and mindfulness [27], neurodevelopmental disorders [28], or childhood emotional abuse [29,30]) and PIU. Clinically, dissociation serves as a psychological defense mechanism where individuals detach from their current awareness. In the context of PIU, this potentially allows for the immediate avoidance of distressing emotions, for example those related to loneliness, by immersing in the virtual world [31]. This detachment could facilitate prolonged Internet engagement by reducing awareness of time and self [27,32]. Nevertheless, it may also exacerbate the cycle of loneliness and PIU, making it crucial to examine dissociation’s impact on therapeutic and preventive strategies for PIU.

Given the consistent body of research separately connecting loneliness and dissociation with PIU, it seems necessary to improve the understanding of how loneliness impacts PIU in relation to mechanisms of dissociation. The relation between loneliness and dissociation in connection to PIU has indeed been considered only in one study, which showed that college students with high levels of loneliness had significantly higher scores for measures of online dissociation than their colleagues with low levels of loneliness [33].

It seems also fundamental to perform this kind of research in adolescents as it is recognized that loneliness and PIU in this age group are of diffuse concern [3,9,34,35]. To better comprehend the current manifestation of PIU in adolescents, two factors appear to be relevant. The first is the pervasive employment of Internet-based communication technologies in daily lives, in particular starting from the early 2010s with the advent of smartphone-based social media. During this period, research has also observed a rise in levels of loneliness among adolescents [34,35]. The second is represented by the psychological peculiarities of adolescence [36], in particular regarding heightened sensitivity to social feedback, as well as increased emotional reactivity, and the ongoing development of self-regulation skills [37]. These characteristics make adolescents particularly susceptible to developing PIU as they seek social validation and connection through online platforms while they cannot rely on fully developed emotional and self-regulatory skills [38]. Moreover, it should also be noted that, albeit not necessarily resulting from a pathological condition, various dissociative symptoms, such as absorption, imaginative involvement, depersonalization, amnesia, derealization, or disturbance in identity, may be more common in adolescents than in adults [39,40]. This predisposition may facilitate increased engagement of adolescents in virtual worlds, self-identification with avatars, and difficulty in differentiating between Internet-mediated and in-person communication.

In the present study, a sample of high school adolescent students was included, and measurements of PIU, loneliness, and dissociation were collected. This study aimed to analyze the mutual relationship between PIU, loneliness, and dissociation, in particular by investigating the potential mediating role of dissociation in the relationship between loneliness and PIU.

## 2. Materials and Methods

### 2.1. Procedure

An anonymous online survey was administered to high school students during school hours, spanning from January to June 2020. Part of the data (38.3%) were collected up to February 21, before the interruption of school activities due to the outbreak of the COVID-19 pandemic; the rest (61.7%) were collected using online forms (using the Google Forms platform, https://www.google.com/forms/about/, accessed on 22 October 2024) when school activities had restarted with online lessons. The survey was conducted across eight schools located in the city of Udine, Italy. All adolescents who expressed their willingness to participate were included in the study after obtaining informed consent from their parents. No incentives were provided to students for their participation in the study.

The current research was approved by the Institutional School Boards of the schools involved in the project from 25 October 2019 to 25 January 2020, in agreement with the University of Udine, the local public health agency (ASUFC), and the Municipality of Udine.

### 2.2. Participants

Two hundred and forty-three high school students (69.1% females, 30.9% males) were included in the study. Data from five students were excluded for missing responses in the survey. Characteristics of the study participants are presented in Table 1. The majority of the study participants (72.4%) attended the lyceum high school, while the others attended the professional high school (14.8%) or the technical high school (12.8%). The sample included students from all five years of high school, aged 14 to 20 years (M = 17.1, SD = 1.4). Most of the participants practiced sports (58.4%) and engaged in a hobby (90.9%).

### 2.3. Measures

PIU was assessed using the Generalized Problematic Internet Use Scale-2 (GPIUS-2) [4] in its Italian version [41]. This is a 15-item tool, in which respondents rate the degree of agreement with each statement (example items: “1. I prefer online social interaction over face-to-face communication”; “5. My internet use has made it difficult for me to manage my life”) on an 8-level Likert scale (from 1 = “definitely disagree” to 8 = “definitely agree”). The GPIUS-2 provides scores in four subscales (preference for online social interactions, mood regulation, deficient self-regulation, and negative outcomes) and a total score (range 15–120) that was used in the present study, with higher scores indicating higher PIU. A construct validity assessment in the original Italian study showed a better fit for a four-factor than for a single-factor model [41], but the total score is commonly used in research. The reliability of the scale in the present study was found to be satisfactory (Cronbach’s alpha = 0.89).

Loneliness was assessed with the UCLA Loneliness Scale version 3 (UCLA-LS) [42] in its Italian translation. Using a 4-level Likert scale (from 1 = “never” to 4 = “always”), respondents answer 20 questions regarding how often they feel disconnected from others (example items: “2. How often do you feel that you lack companionship?”; “4. How often do you feel alone?”). The UCLA-LS provides a total score (range 20–80), with higher scores indicating greater feelings of loneliness. The construct validity assessment in the original study provided support for the unidimensionality of the scale [42]. The reliability of the scale in the present study was found to be excellent (Cronbach’s alpha = 0.92).

Dissociation was assessed using the Adolescent Dissociative Experiences Scale (A-DES) [43] in its Italian version [40]. In this 30-item tool (example items: “13. I don’t recognize myself in the mirror”; “25. I find myself standing outside of my body, watching myself as if I were another person”), respondents rate the frequency of their dissociative experiences using an 11-point Likert scale (from 0 = “never” to 10 = “always”). The scale provides scores in four subscales (dissociative amnesia, absorption and imaginative involvement, depersonalization and derealization, passive influence) and a total score (range 0–300) that was used in the present study, with higher scores indicating a higher frequency of dissociative experiences. Construct validity assessment in the original Italian study showed an acceptable fit for the single-factor model [40]. The reliability of the scale in the present study was found to be excellent (Cronbach’s alpha = 0.94).

Additionally, socio-demographic variables including age, gender, type of school (lyceum/technical high school/professional high school), school year (9th-13th), participation in sports (yes/no), and hobby engagement (yes/no) were collected.

### 2.4. Statistical Analyses

In this cross-sectional study, descriptive statistics for PIU scores were initially provided. A GPIUS-2 cut-off score to identify severe PIU was derived from the study of Machimbarrena and colleagues [44], where a GPIUS-2 cut-off score of 52 for severe PIU in adolescents was obtained by employing the GPIUS-2 scale with a 6-level Likert response scale. In the current study, a GPIUS-2 cut-off score of 69 for severe PIU was derived proportionally.

To analyze whether socio-demographic variables (age, gender, type of school, sport practice, hobby engagement) and time of assessment (before/during the COVID-19 pandemic) were associated with PIU, a multiple linear regression analysis was performed. School year was not included in the analysis because of the collinearity with age. Categorical predictor variables were coded as follows: gender (0 = female, 1 = male), type of school (1 = lyceum, 2 = technical high school, 3 = professional high school), sport practice (0 = no, 1 = yes), hobby engagement (0 = no, 1 = yes), and time of assessment (0 = before the COVID-19 pandemic, 1 = during the COVID-19 pandemic).

Relationships between GPIUS-2, UCLA-LS, and A-DES scores were evaluated through Pearson’s correlation analysis.

Finally, a mediation analysis was performed to explore the indirect effect of A-DES scores on the association between UCLA-LS and GPIUS-2 scores. This analysis was conducted using the Process macro [45], applying Model 4 with bootstrapping (10,000 samples), with socio-demographic variables and time of assessment (before/during the COVID-19 pandemic) serving as covariates. These variables were coded as described above for the regression model on PIU.

The analyses were performed with the free software environment R, version 3.6.3. All effects are reported as significant at *p* < 0.05.

## 3. Results

PIU was either absent or mild in 222 students (91.4% of the sample) and severe in 21 students (8.6% of the sample). With regression analysis, where the model met the assumptions of no perfect multicollinearity (all Variance Inflation Factors, VIFs, were between 1.06 and 1.39) and independence of errors (Durbin–Watson statistic = 2.01, *p* = 0.923), it was shown that none of the socio-demographic variables or the time of assessment (before/during the COVID-19 pandemic) significantly predicted PIU (for all predictors, *p* ≥ 0.07, see Table 2).

As shown in Table 3, correlation analyses indicated that higher UCLA-LS and A-DES scores were significantly associated with higher GPIUS-2 scores. In particular, UCLA-LS positively correlated with GPIUS-2 (r = 0.264, *p* < 0.001), A-DES positively correlated with GPIUS-2 (r = 0.436, *p* < 0.001), and UCLA-LS positively correlated with A-DES (r = 0.448, *p* < 0.001).

**Figure 1:**
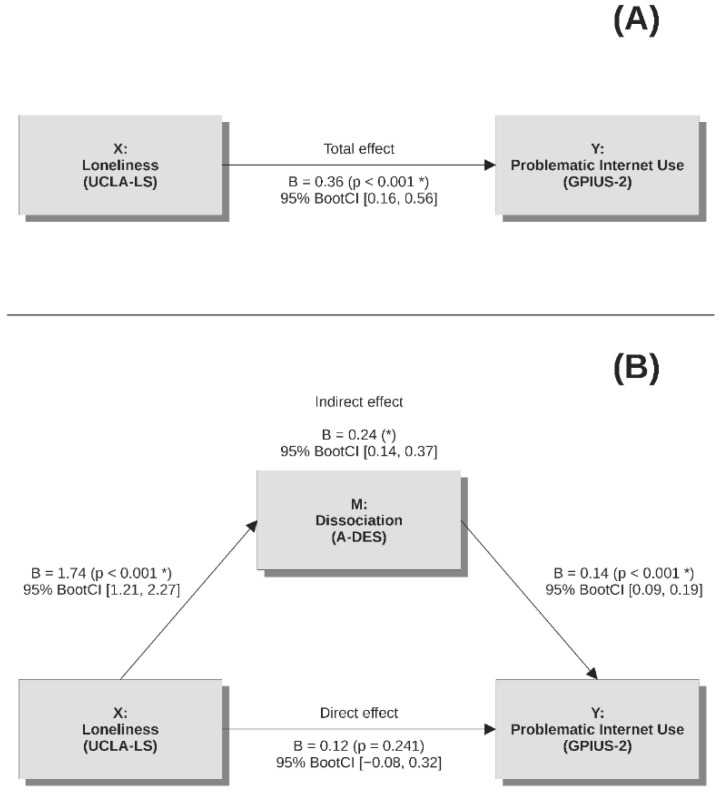
Simple mediation by dissociation in the association between loneliness and problematic Internet use. (**A**) Regression model of loneliness (predictor) and problematic Internet use (outcome); (**B**) mediation model of loneliness (predictor), dissociation (mediator), and problematic internet use (outcome). Note: covariates were entered in the models (gender: female/male; type of school: lyceum/technical high school/professional high school; school year: 9th–13th; sport practice: no/yes; hobby engagement: no/yes; time of assessment: before/during the COVID-19 pandemic). Abbreviation and symbols: A-DES = Adolescent Dissociative Experiences Scale, B = unstandardized regression coefficient, BootCI = Bootstrap Confidence Interval, GPIUS = Generalized Problematic Internet Use Scale, UCLA-LS = University of California Los Angeles – Loneliness Scale, * = *p* < 0.05.

The results of the mediation analysis are summarized in Figure 1. The initial regression model (Figure 1, panel A), where predictors explained 9.9% of the variance in GPIUS-2 scores (F(8234) = 3.2, *p* = 0.002), highlighted a significant total effect of UCLA-LS on GPIUS-2 scores (B = 0.36, SE = 0.10, t = 3.6, *p* < 0.001, 95% Boot CI [0.16, 0.56]; β = 0.24). When A-DES scores were added in the model and dissociation was considered a potential mediator (Figure 1, panel B), the model accounted for 22.1% of the variance in GPIUS-2 scores (F(9233) = 7.4, *p* < 0.001). UCLA-LS predicted A-DES scores (B = 1.74, SE = 0.27, t = 6.5, *p* < 0.001, 95% Boot CI [1.21, 2.27]; β = 0.40) and A-DES predicted GPIUS-2 scores significantly (B = 0.14, SE = 0.02, t = 6.0, *p* < 0.001, 95% Boot CI [0.09, 0.19]; β = 0.40). Moreover, the previously significant direct effect of UCLA-LS on GPIUS-2 scores became non-significant (B = 0.12, SE = 0.10, t = 1.2, *p* = 0.240, 95% Boot CI [−0.08, 0.32]; β = 0.08), while the indirect effect via A-DES scores was significant (B = 0.24, SE = 0.06, 95% Boot CI [0.14, 0.37]; β = 0.16). The indirect effect was significantly different from zero, because the 95% Boot CI did not include zero. In the three regression models employed in the mediation analysis, all covariates (socio-demographic variables and time of assessment) had a non-significant effect on model outcome scores (|t| < 1.8, *p* > 0.069).

## 4. Discussion

The current study aimed to explore adolescents’ problematic Internet use (PIU) and its associations with loneliness and dissociation. The first result is about the prevalence of severe PIU, which amounted to 8.6% of the sample. Although there is variability across the studies in prevalence estimates of PIU [46,47], probably also due to the lack of overall consensus on the definition of PIU, the employment of different assessment tools, and the different times and locations of studies, the result obtained in the present study is consistent with many of the previous research estimates of PIU, generally ranging from 5% to 10% of the general population [48,49]. It is important to note that the measure of PIU employed in the present study (i.e., the GPIUS-2 questionnaire) did not originally include a cut-off score for severe levels, but a cut-off was derived from a previous study on adolescents (n = 12,285) which employed latent profile analysis on self-rated scores of health-related quality of life [44]. When considering the raw GPIUS-2 scores observed in the current study conducted in 2020, a comparison with scores collected at other time points from similar samples (i.e., Italian high school students) showed that the GPIUS-2 scores from the current study (M = 43.7) were higher than those collected during the validation study of the Italian version of the GPIUS-2 in 2013 (M = 34.8, n = 242) [41]; they were similar to those collected in a study published in 2021 (M = 42.2, n = 766) [50] and lower than those collected in a study published in 2024 (M = 47.8, n = 574) [51]. Research conducted also beyond the Italian context has indeed observed an increase in PIU over the past decade [52,53]. According to surveys from international agencies, PIU and loneliness in Italian adolescents measured before and after the current study (2020) appeared in line with the average international levels [53,54,55].

This study then examined through regression analysis the association between PIU and some common socio-demographic and situational factors, i.e., gender, age, type of school, sport practice, hobby practice, and assessment before/during the COVID-19 pandemic. The results indicated that none of these variables significantly predicted PIU. Regarding the non-significant association of PIU with age and gender, it is worth underlying that previous research in adolescents has not consistently identified an association between these variables and PIU [15,20,56]. Moreover, most of the studies on PIU during the beginning of the COVID-19 pandemic did not find significant differences in PIU levels measured immediately before vs. in the first months of the COVID-19 pandemic [49], and, in the current study, assessment time relative to the COVID-19 pandemic did not significantly predict observed PIU levels.

When considering PIU in relation to loneliness and dissociation, the present study first confirmed that, in adolescents, more frequent feelings of loneliness and a higher dissociative tendency were associated with higher PIU [24,57,58]. These three variables were then subjected to mediation analysis, when controlling for socio-demographic and contextual variables. In this analysis, an indirect effect of loneliness on PIU through dissociation was observed. This suggests, at least within the current study sample and for the variables taken into account as covariates, that dissociation may serve as a significant link between loneliness and PIU. In particular, higher loneliness predicted greater dissociation, which in turn was predictive of higher PIU.

This result builds upon and complements prior research linking psychological variables to PIU via the mechanism of dissociation. Schimmenti et al. [26] observed an indirect effect of dissociation on the relationship between attachment disorganization and Internet addiction scores in a sample of adult online gamers with significant symptoms of PIU.; Mazzoni et al. [27] showed that temporal dissociation mediated the effect of self-control and mindfulness on PIU in young social media users; and Verrastro et al. [30] reported an indirect effect of dissociation on the association between childhood emotional abuse and PIU in a sample of high school adolescents. The current study seems, therefore, relevant to this literature for focusing on dissociation and PIU together with loneliness. The experience of loneliness can arguably lead to a heightened sense of disconnection from one’s surroundings and self, prompting adolescents to turn to the Internet as a means of escape. This dissociative behavior may reinforce their PIU, creating a vicious cycle where the more they feel lonely, the more they dissociate and rely on the Internet, ultimately worsening their PIU.

The main finding of the current study is interesting, especially given the prevalent mental health challenges faced by adolescents [59], compounded by the widespread engagement in Internet-based activities. They are in line with current indications on the importance of equipping adolescents with preventive and therapeutic tools to raise awareness about themselves, life, and the potential risks and opportunities of the virtual world, as well as to alleviate excessive dissociative tendencies [60] and feelings of loneliness [61]. For loneliness, older studies suggested that most effective interventions addressed maladaptive social cognition [62]. Campaigns aimed at increasing awareness of loneliness and diminishing its associated stigma have been implemented in numerous countries [63]. In schools, where fostering positive social environments in the classroom is essential in addressing loneliness [64], prevention programs have the potential to be cost-effective and can reach the entire population at risk [65]. As a practical application of the current research in clinical interventions, campaigns, schools, and in other educational contexts, the mechanism of dissociation should be taken into account when designing interventions for the reduction in loneliness in adolescents, also in relation to PIU. This task could be accomplished both by providing adolescents with information on dissociation and on its links with loneliness and PIU, and by endowing them with the abilities to recognize and cope with dissociative experiences, thereby minimizing the potential negative outcomes on activities such as Internet use.

With regard to limitations, the present study employed a relatively small sample size, which may affect the generalizability of the findings. Additionally, this study analyzed the potentially bidirectional interactions between loneliness and PIU only in a unidirectional way, not accounting for the possibility that PIU could also lead to increased feelings of loneliness. Moreover, the mediation model included only the variable of dissociation, while other variables could concurrently mediate the effects of loneliness on PIU. Finally, self-report measures, while valuable, can introduce bias due to social desirability or inaccurate self-assessment. Future research should consider larger, more diverse samples, wider sets of mediators, and employ longitudinal designs to better understand the temporal dynamics and causal relationships between these variables.

## 5. Conclusions

This study showed an indirect effect of dissociation on the relationship between loneliness and PIU in an adolescent sample, with positive associations between loneliness and PIU, loneliness and dissociation, as well as between dissociation and PIU. Given the observed mediating role of dissociation in this context, it seems crucial for future interventions to address both loneliness and dissociative tendencies to effectively mitigate PIU and its associated mental health impacts in adolescents. Expanding our understanding of these interactions can inform the development of more comprehensive prevention and treatment strategies tailored to the psychological needs of adolescents. This task seems very relevant, based on the current tendency of conducting social interactions through the medium of Internet-based technologies and on the growing concern about the impact of these technologies on adolescent mental health.

## Figures and Tables

**Table 1:** Characteristics of study participants (n = 243).

Variable	Level	M ± SD, or n (%)
Age		17.1 ± 1.4
Gender	Female	168 (69.1%)
	Male	75 (30.9%)
Type of high school	Lyceum	176 (72.4%)
	Technical	31 (12.8%)
	Professional	36 (14.8%)
School year	9th	44 (18.1%)
	10th	17 (7.0%)
	11th	50 (20.6%)
	12th	96 (39.6%)
	13th	36 (14.8%)
Sport practice	No	101 (41.6%)
	Yes	142 (58.4%)
Hobby	No	22 (9.1%)
	Yes	221 (90.9%)
Assessment time	Before the COVID-19 pandemic	93 (38.3%)
	During the COVID-19 pandemic	150 (61.7%)

**Table 2:** Multiple linear regression model for PIU (GPIUS-2 total score).

Predictor	B	SE	t Score	*p* Value
Age	−1.64	0.94	−1.76	0.081
Gender	−3.07	2.49	−1.23	0.220
Type of high school	2.66	1.66	1.61	0.109
Sport practice	−4.44	2.46	−1.80	0.073
Hobby	−0.52	4.03	−0.13	0.898
Assessment time	2.22	2.72	0.82	0.415

Abbreviations: B = unstandardized regression coefficient, SE = Standard Error of B.

**Table 3:** Descriptive statistics and correlation matrix of the scales.

Score	M (SD)	Skewness (SE) Kurtosis (SE)	Cronbach Alpha	1.	2.	3.
1.GPIUS-2 total (PIU)	43.7 (18.0)	0.813 (0.156) 0.850 (0.311)	0.88	1.000		
2.UCLA-LS total (Loneliness)	46.1 (11.8)	0.254 (0.156) −0.471 (0.311)	0.92	0.264 ***	1.000	
3.A-DES total (Dissociation)	70.2 (51.1)	0.892 (0.156) 0.211 (0.311)	0.94	0.436 ***	0.448 ***	1.000

Abbreviations and symbols: A-DES = Adolescent Dissociative Experiences Scale, GPIUS = Generalized Problematic Internet Use Scale, M = Mean, SD = Standard Deviation, SE = Standard Error, UCLA-LS = University of California Los Angeles—Loneliness Scale, *** = *p* < 0.001.

## Data Availability

The data that support the findings of this study are available on request from the corresponding author, A.M. The data are not publicly available as part of an ongoing study.
